# Estimating the perceived dimension of psychophysical stimuli using triplet accuracy and hypothesis testing

**DOI:** 10.1167/jov.22.13.5

**Published:** 2022-12-05

**Authors:** David-Elias Künstle, Ulrike von Luxburg, Felix A. Wichmann

**Affiliations:** 1 Department of Computer Science, University of Tübingen, Germany

**Keywords:** psychophysics, psychophysical scaling, multi-dimensional scaling, MDS, ordinal embedding, dimensions, triplet, triad, hypothesis test, accuracy

## Abstract

Vision researchers are interested in mapping complex physical stimuli to perceptual dimensions. Such a mapping can be constructed using multidimensional psychophysical scaling or ordinal embedding methods. Both methods infer coordinates that agree as much as possible with the observer’s judgments so that perceived similarity corresponds with distance in the inferred space. However, a fundamental problem of all methods that construct scalings in multiple dimensions is that the inferred representation can only reflect perception if the scale has the correct dimension. Here we propose a statistical procedure to overcome this limitation. The critical elements of our procedure are i) measuring the scale’s quality by the number of correctly predicted triplets and ii) performing a statistical test to assess if adding another dimension to the scale improves triplet accuracy significantly. We validate our procedure through extensive simulations. In addition, we study the properties and limitations of our procedure using “real” data from various behavioral datasets from psychophysical experiments. We conclude that our procedure can reliably identify (a lower bound on) the number of perceptual dimensions for a given dataset.

## Introduction

Some things feel more similar than others: Violet is bluish and reddish but not greenish, trumpet and trombone do not sound the same but are different from the violin, and platinum appears more similar to silver than gold.

One popular idea is that perceived similarities—for example, similar colors, sounds from musical instruments, or materials—correspond with distances in a coordinate system in the perceiver’s mind. Methods allowing to infer the distances and the dimensionality of the internal perceptual space may, thus, be helpful for scientists attempting to understand perception.

Studies about lightness perception find, for example, that the corresponding perceptual space is not necessarily one dimensional (Umbach, 2014; Schmid & Anderson, 2017); human observers are able to disentangle dimensions for the surface color and the illumination (Logvinenko & Maloney, 2006). Material perception research suggests that the perceptual space of materials may be spanned by subjective material properties like softness, viscosity, reflectance, or translucency (Fleming, 2017). Perceived gloss has been found to not only depend on the (physical) specular reflectance of the material, but to also increase with the bumpiness of the surface (Ho, Landy, & Maloney, 2008; Kim, Marlow, & Anderson, 2011; Marlow, Kim, & Anderson, 2011, 2012). Recently, attempts have even been made to estimate the dimensions and the overall (high) dimensionality of object perception from large-scale crowd-sourcing studies (Hebart, Zheng, Pereira, & Baker, 2020; Love & Roads, 2021; Roads & Love, 2021). In all these examples, the dimensions of perceptual experience are of interest—the psychophysical scale, as it is classically and frequently also referred to (see Gescheider, 1988, 2013a, 2013b, for an overview).

The oldest and most frequently used scaling algorithm for more than one dimension is (nonmetric) *multidimensional scaling* (MDS) (Shepard, 1962; Kruskal, 1964a, 1964b)), which was recently accompanied by *ordinal embedding* methods from machine learning (Roads & Mozer, 2019; Haghiri, Wichmann, & von Luxburg, 2020). In contrast with other scaling approaches, such as the popular maximum-likelihood difference scaling (MLDS) (Knoblauch & Maloney, 2012), MDS and ordinal embedding can estimate multiple perceived dimensions. An approach related to MLDS but for multiple dimensions, maximum-likelihood conjoint measurement (Ho et al., 2008), tries to obtain interpretable dimensions by additional assumptions (e.g. monotonicity and independence of perceived dimensions; Radonjić, Cottaris, & Brainard, 2019), whereas MDS and ordinal embedding is more exploratory in trying to find the scale that best fits the data.

MDS estimates the scale by minimizing a *stress* term, measuring the agreement between the distances in the scale and dissimilarity ratings collected for (all) stimulus pairs in a psychophysical experiment.

In contrast with MDS, ordinal embedding methods use triplet comparison judgments of the form “is stimulus A more similar to B or C?” to estimate the scale (Haghiri et al., 2020). This triplet judgment task is often more intuitive for observers than other scaling tasks (Aguilar, Wichmann, & Maertens, 2017). Furthermore, it has to be performed on just a fraction of all possible comparisons, making ordinal embedding methods feasible with more stimuli than MDS (Haghiri et al., 2020).

**Figure 1.**
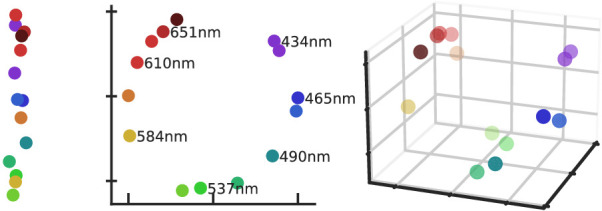
The same perceived hue similarities are represented in a one-, a two-, and a three-dimensional representation. The two-dimensional scale accurately represents the similarities in a circular structure. However, the one-dimensional scale violates some obvious similarities (e.g., orange is distant to red); the three-dimensional scale is too complex because the vertical offsets carry little to no perceptual information. The distances are based on similarity ratings between stimuli of different wavelengths (Ekman, 1954), the colors used for illustration are RGB approximations and differ from the original stimuli.

However, one fundamental problem of psychophysical scaling in multiple dimensions is choosing the “correct” number of dimensions, because both scaling methods, MDS and ordinal embedding, require the user to specify the dimensionality as a method parameter. Unfortunately, the scale’s representation can only reflect perception if it has the “correct” dimensionality. The problem is illustrated in Figure 1 using perceived color similarities. The very well-known color circle is obtained only in a two-dimensional embedding. The distances in the one-dimensional embedding are distorted, so conclusions about the perceived similarities are misleading. Although the distances are correct in the three-dimensional embedding, the additional third dimension carries no perceptually valid information and is thus also misleading.

Hence, dimensionality is crucial in multidimensional perceptual scaling. The standard approach to choosing the “correct” the dimensionality for MDS is visualizing the stress for different dimensions—ideally, stress should decrease and show a knee at the intrinsic (correct) dimensionality (Borg & Groenen, 2005). Perceptual studies with ordinal embedding algorithms used various approaches to determine the correct dimensionality. Some studies first estimated a high dimensional scale with an ordinal embedding algorithm and subsequently dropped dimensions until a measure of explained variance fell below a predefined threshold (Toscani, Guarnera, Guarnera, Hardeberg, & Gegenfurtner, 2020). Others selected the dimension where the probability of hold-out judgments, namely judgments that were not used to estimate the scale, is maximal (Roads & Love, 2021) or where the judgement accuracy is beyond an (arbitrary) threshold (Haghiri et al., 2020).

However, none of these attempts to infer the appropriate or correct dimensionality is entirely satisfactory. There is a highly subjective component in inspecting a stress graph or choosing the variance or accuracy threshold. In addition, these methods do not explicitly consider the intrinsic stochasticity of the perceptual judgments (random sampling, human factor) and the stochasticity of scaling algorithms themselves (random initialization). Thus, an appropriate dimension estimation procedure should include the distribution or variation of the scale’s accuracy and provide an interpretable decision criterion—a typical application of a statistical test. Such tests have already been applied to scaling models; for example Radonjić et al. (2019) use a t-test on the cross-validated model fit of an maximum-likelihood conjoint measurement-like scaling model to decide between the Euclidean or city-block distance metrics.

Here we propose a statistical procedure inspired by model selection to choose the dimensionality: Tuning the dimensionality can prevent underfitting and overfitting. Too simple models do not fit the data well enough; conversely, too complex models can typically fit the data but are prone to overfitting, that is, fitting noise instead of behavior. This view transforms the dimensionality choosing problem into a model selection problem—and allows us to benefit from the extensive and time-proven model selection literature and methods. The critical elements of our suggested dimensionality estimation procedure are, first, measuring the scale’s quality by the number of correctly predicted triplets (cross-validated triplet accuracy); second, performing a statistical test to assess if adding another dimension improves triplet accuracy significantly. To validate this procedure, we simulated noisy and sparse judgments and assessed reliability in identifying the ground-truth dimensionality. Furthermore, we studied the properties and limitations of our procedure using “real” data from various behavioral datasets from psychophysical experiments.

We conclude that our procedure is a robust tool for exploring new perceptual spaces and can help identify a lower bound on the number of perceptual dimensions for a given dataset.

## Scaling, procedure, and simulations

This section first introduces the fundamental concepts of triplets, ordinal embedding algorithms, and triplet accuracy; afterward, it describes our procedure for dimension estimation and shows results from simulations for validation.

### Background: Triplets and ordinal embedding

#### Triplets reflect stimulus similarities

Psychophysical scaling attempts to create a geometric, distance-based representation of perceived (stimulus) similarity. Similarity can be measured by many different experimental tasks of which the *triplet* (or triad) task is reasonably common (Wills, Agarwal, Kriegman, & Belongie, 2009; Devinck & Knoblauch, 2012; Bonnardel et al., 2016; Lagunas et al., 2019; Haghiri et al., 2020; Toscani et al., 2020). In the triplet task, observers are presented with three different stimuli, usually simultaneously, of which one is called the *anchor*. The observer chooses one of the other two stimuli perceived as most similar (or dissimilar) to the anchor, resulting in the (*anchor, near, far*)—a triplet of stimulus indices. The triplet task comes in different flavours depending on the instructions, for example, “Which is the odd one out?” (Hebart et al., 2020); or the opposite question “Which appears most central?” (Kleindessner & von Luxburg, 2017). Sometimes observers are presented with more stimuli and are asked to make multiple decisions, as, for example, in (Roads & Mozer, 2019; Roads & Love, 2021). From an ordinal embedding perspective, these differences are irrelevant—the responses can be mapped to triplets (interested readers can find details about the mappings in the Supplementary Material A).

Collecting more data (triplets) will lead to more accurate results corresponding with a more accurate psychophysical scale in the context of scaling or ordinal embedding. Furthermore, the required number of trials in a triplet experiment also depends on the number of stimuli n—which is known—and the dimensionality d of the stimulus space—which is unknown. As a rule of thumb (Haghiri et al., 2020) recommend to use at least $2dn\log_{2}n$ triplets. This rule is based on a mathematical proof about the number of triplets required to reconstruct the scale up to a small error (Jain, Jamieson, & Nowak, 2016). Because of the proof, we know that often a fraction of the possible 3n3 triplets is sufficient to reconstruct the scale and that the number of trials must increase with both the perceived dimension and the number of stimuli.

In practice, there are very different triplet-based experiments, although their methodological and statistical choices are important, almost always, the choice of stimuli might be even more critical. Some laboratory-based experiments present fewer than 100 stimuli in several hundred or few thousand triplets (e.g., Aguilar et al., 2017; Toscani et al., 2020), whereas crowd-sourced online experiments present up to 50,000 stimuli in millions of trials (e.g., Hebart et al., 2020; Roads & Love, 2021). Typically, these triplets show distinguishable stimuli, that is, the differences are suprathreshold, but similar enough for “reasonable” comparisons: variations or changes of material properties or samples from the same domain (e.g., images of landscapes). Otherwise, answering the comparisons might become challenging or the comparisons measure cognitive associations instead of perception: The question, “What is more similar to a tree, the sun or a neuron?” could be judged based on concepts like photosynthesis or the tree-ish look of the neuron’s dendrites. Data from such experiments can be embedded into the similarity space, but are unlikely to yield insights into the workings of the visual system.

#### Ordinal embedding methods estimate scales

Psychophysical scales represent the stimuli as coordinates in d dimensions, whose distances should correspond to the perceived stimulus similarity. Ordinal embedding algorithms choose these coordinates $\psi_{1},\cdots ,\psi_{n}\in R^{d}$ by maximizing the *agreement* between triplets (*anchor, near, far*) and the corresponding distances in terms of Equation 1. Similarly, we call triplets where the inequality does not apply *disagreeing triplets*.

(1) $$\begin{array}{c} \mathrm{dist}\left(\psi_{\mathrm{near}},\psi_{\mathrm{anchor}}\right)\leq \mathrm{dist}\left(\psi_{\mathrm{anchor}},\psi_{\mathrm{far}}\right).\; \end{array}$$

The coordinate estimation requires no stimulus attributes or neighborhoods and, provided enough data, provably recovers metric information up to similarity transformations (translation, rotation, reflection, scaling), and a small error (Kleindessner & Luxburg, 2014; Jain et al., 2016)—assuming appropriate dimension and distance metrics. The appropriate distance metrics of psychological spaces are actively discussed (for recent discussion, see, e.g., Logvinenko & Maloney, 2006; Love & Roads, 2021), but the standard Euclidean distance is perhaps the most intuitive and the most commonly used; thus, we use it here.

Algorithmically, ordinal embedding methods optimize coordinates that minimize a *stress*-function on a set of triplets $T=\{\left(i_{1},j_{1},k_{1}\right),\cdots ,\left(i_{m},j_{m},k_{m}\right)\}$, where i,j,andk are stimulus indices whose order correspond with the trial response *anchor*, *near*, and *far*. The numerical properties of this stress-function (e.g., smoothness) are more desirable for optimization than the agreement count (Equation 1). Different ordinal embedding algorithms mainly differ in their stress-function (Agarwal et al., 2007; van der Maaten & Weinberger, 2012; Terada & Luxburg, 2014; Jain et al., 2016). The algorithm that we use in this work is called *soft ordinal embedding* (Terada & Luxburg, 2014) and has been shown to result in very accurate reconstruction in a large scale benchmarking study (Vankadara, Haghiri, Lohaus, Wahab, & von Luxburg, 2020). Soft ordinal embedding's stress function (Equation 2) is “soft” in the sense that disagreeing triplets are included based on the size of their squared error. Trivial solutions with all-zero coordinates are prevented by enforcing a minimal distance difference (“⋯+1”). Once a coordinate triplet agrees by this minimal distance it does not increase the stress (“max[0,⋯]”).

(2) $$\begin{array}{c} \;\;\;\;\;\;\;\;\;\;\;\sum_{(i,j,k)\in T}\max\left[0,\mathrm{dist}\left(\psi_{j},\psi_{i}\right)-\mathrm{dist}\left(\psi_{i},\psi_{k}\right)+1\right]{}^{2}.\; \end{array}$$

Equation 2 is minimized for coordinates ψ by the Broyden–Fletcher–Goldfarb–Shanno algorithm (see Fletcher, 1987). Unfortunately, the optimization is nonconvex and sometimes converges to suboptimal solutions. Thus, each optimization is restarted from ten random initializations, returning the scale with minimal stress.

#### Triplet accuracy measures the scale’s fit

Although the stress is useful to maximize the scale’s fit on training triplets, it is not a good indicator of the scale’s fit. The stress cannot predict how well the scale matches the observer’s responses *in general*. A more direct measure of fit is the proportion of triplets that agree in terms of Equation 1 with the scale ψ, called the *triplet accuracy* (Equation 3).

(3) $$\begin{array}{c} \;\;\;\;\;\;\;\;\;\;\mathrm{acc}\left(\psi ,T\right)=\frac{1}{m}\sum_{(i,j,k)\in T}1_{\left[\mathrm{dist}\left(\psi_{j},\psi_{i}\right)\leq \mathrm{dist}\left(\psi_{i},\psi_{k}\right)\right]}.\; \end{array}$$

The indicator function $1_{\left[\cdots \right]}$ returns 1 for agreement and 0 otherwise, such that  acc (ψ,T) ranges between 0 (full disagreement) and 1 (full agreement).

The triplet accuracy can be calculated either on the same triplets used to fit the scaling algorithm or on a separate set from the same population to test the scale. Thus, we can distinguish between *training triplet accuracy* (train accuracy) and *test triplet accuracy* (test accuracy). In contrast with the training accuracy, the test accuracy helps to distinguish reasonable from *noisy* responses. Noise in the sensory system, lapses, or other human imperfections, summarized as *judgment noise*, might cause erroneous triplets that disagree with the majority of responses. However, the test triplets likely contain different erroneous triplets; this process results in a lower test accuracy that is a better estimate of the general or “true” fit than the (spuriously high) training accuracy.

#### Cross-validating test accuracy

Triplet collection in a perceptual context is time consuming. Thus, instead of collecting entirely disjunct training and test data, we advocate using a resampling algorithm for a data-efficient approximation of the test accuracy. This resampling-algorithm, *k-fold cross-validation* (compare Hastie, Tibshirani, & Friedman, 2009), splits the dataset into k equal-sized parts and estimates k scales to calculate k accuracies. For every iteration, k-1 different parts are used for training, while the k-th part is used for testing the accuracy. To sample more than k accuracies, *r-repeated cross-validation* repeats the k folds on r shuffled versions of the dataset. The mean of the resulting k·r accuracies approximates the test accuracy. Please note that these cross-validated scales are used for accuracy estimation—the “final” scale for visualizing the observer’s perception is estimated from all triplets.

#### Trading off dimensions against accuracy

Psychophysical scales can be seen as parametric models whose parameters are the stimulus coordinates in the perceptual space. Like any parametric model, scales, too, are affected by underfitting and overfitting if coordinates have too few or too many dimensions. We illustrate this by revisiting the example from the introduction (Figure 1). One can imagine triplets encoding the distance relations in the two-dimensional scale. The one-dimensional scale lacks sufficient freedom to capture all triplets; the scale is *underfitting*. For example, red is perceived as more similar to yellow than blue; in the one-dimensional fit, red is farther from yellow than blue. The three-dimensional scale might perfectly represent all triplets, but also all the erroneous ones (zero training accuracy)—the scale is *overfitting*. The erroneous triplets, caused by judgement noise, disagree with most triplets and thus with the most accurate scale.

### Our procedure: Testing for accuracy gains

Following the previous considerations of underfitting and overfitting for different scaling dimensions, a suitable procedure should choose the dimension in which the test accuracy is maximal. However, given the noise inherent in psychophysical data—and thus in scale estimates and accuracies—a purely visual inspection will not do. We require a statistical test for dimensionality based on the triplet accuracy.

#### Related dimension estimation methods

The statistics and machine learning literature proposes several dimension estimation methods, but they are unsuitable for analyzing psychophysical data.

The vast majority of methods in machine learning use metric data (see Camastra & Staiano, 2016, for an overview); that is, every data point is described by a collection of numerical features. However, data from perceptual scaling experiments like rankings or triplets only provide the order of stimulus similarities.

Only a few dimension estimation methods use nonmetric data. However, they all require more data than we typically can collect in psychological experiments: The method of Kleindessner and Luxburg (2015) estimates the dimensionality from information about the k nearest neighbors of each datapoint (e.g., the k most similar stimuli).

However, it is not straightforward to calculate the k nearest neighbors from triplets. Additionally, the method’s performance highly depends on the number of objects, which are the stimuli in our setting; the authors tested their method with $5\cdot 10^{4}$ to $5\cdot 10^{7}$ objects, which is far from a feasible stimulus size.

The other nonmetric dimension estimation method that we are aware of follows another approach, but is similarly difficult to apply in a perceptual setting (Tabaghi, Peng, Milenkovic, & Dokmanic, 2021) derive a so-called *ordinal capacity* metric that differs between metric spaces, for example, one-dimensional Euclidean, two-dimensional Euclidean, and four-dimensional hyperbolic space. Calculating this ordinal capacity requires sorting the distances between n stimuli which requires in the limit up to n times more triplets than estimating a (low-dimensional) scale $O(n^{2}\log n)$ instead of O(dnlogn) with d≪n; (Haghiri et al., 2020). In an exemplary case with a two-dimensional scale of 40 stimuli, one requires approximately 20 times more triplets to determine the ordinal capacity than to estimate the scale. This additional experimental effort—just for determining the dimensionality—is unacceptable.

#### Test for a significant gain in accuracy

The elements of our procedure are statistical tests between scales with increasing dimensionality d and d+1, testing if adding a dimension improves the mean test accuracy μ, with the null hypothesis $H_{0}^{d}:\mu_{d+1}\leq \mu_{d}$ and alternative $H_{1}^{d}:\mu_{d+1}>\mu_{d}$.

In our procedure, accuracy samples ${\mathrm{acc}}_{d},{\mathrm{acc}}_{d+1}\in R^{rk}$ are collected using repeated cross-validation with r repetitions and k folds. By default, we use r=k=10 based on empirical results in the model comparison literature (Bouckaert & Frank, 2004), leading to 100 samples per dimension. The accuracy gain ${\mathrm{acc}}_{d+1}-{\mathrm{acc}}_{d}$, paired by repetition and fold, is evaluated with a two-sample *t* test whose test statistic is modified for the use of cross validation.

A standard *t* test assumes normally distributed and independent samples. Although accuracy samples are binomial and thus approximately normally distributed (see Dietterich, 1998, for the argument and see the Supplementary Material B for simulations), their independence is violated by the data overlap in cross-validation folds. For *t* tests with k-fold cross-validated datasets (Nadeau & Bengio, 2003) proposed the correction factor $\frac{1}{k-1}$ in the test statistic:

(4) $$\begin{array}{c} t=\frac{\mathrm{mean}\left({\mathrm{acc}}_{d+1}-{\mathrm{acc}}_{d}\right)}{\sqrt{\frac{1}{rk}+\frac{1}{k-1}}\cdot \mathrm{sd}\left({\mathrm{acc}}_{d+1}-{\mathrm{acc}}_{d}\right)}.\; \end{array}$$

We use the test statistic to calculate the probability of obtaining the observed accuracy gain under the null hypothesis that there is no gain, the p-value. We accept the accuracy gain only if the p-value is lower than an acceptance threshold α. Beside the modified test statistic, the calculation is identical to a standard one-sided *t* test: The p-value is the probability density of a Student t distribution with rk-1 degrees of freedom at our t-value, calculated with Equation 4.

#### Sequential testing

The goal is to detect the lowest dimension without an accuracy gain in a predefined range—care has to be taken to apply appropriate multiple-testing corrections to prevent the increased risk of false positives. The tested dimension range is the parameter of interest and depends on the perceptual question, namely, the experimental task and stimulus.

In a range of dimensionalities, the procedure tests the neighboring dimensionalities for the alternative hypothesis “gain in accuracy,” then returns the lowest gain-providing dimensionality. If no rejection occurs, the intrinsic dimensionality is assumed beyond the tested range. The more neighboring dimensions are tested, the more likely an erroneous significance occurs (multiple testing problem). The acceptance threshold α should be corrected to compensate for the total number of tests, the best-known correction being the Bonferroni method (Bonferroni, 1936). However, despite its charm of simplicity, the Bonferroni correction is known to overcorrect α once the number of individual tests increases; in other words, the method has low statistical power (Holm, 1979). In practice, we assume that the corrected α of more than three tests would be too small to detect a “gain in accuracy.”

Thus, our procedure uses an improved version of the Bonferroni method with larger statistical power, called the *Holm–Bonferroni method* (Holm’s step-down procedure, Holm, 1979), to correct the significance threshold α of the neighboring dimension tests. The Holm–Bonferroni method is more powerful than basic Bonferroni (fewer false-negative test results) and hardly more complicated, but otherwise shares Bonferroni’s benefits, such as a lack of distributional and dependence assumptions. Whereas the Bonferroni correction divides the significance threshold α by the number of tests m (α corrected =αm) the Holm–Bonferroni correction considers the ascending ranking r of all test’s *p*-values: $\alpha_{r}=\frac{\alpha }{m-r+1}$. The strictest threshold $\frac{\alpha }{m}$ is just used for the smallest p-value, such that fewer “gain in accuracy” tests are erroneously rejected with the Holm–Bonferroni method, although the increase in accuracy usually decreases with increasing dimension.

We became aware that, in pharmaceutical studies, there exists a statistically similar problem, the so-called dose finding problem: The effect of medicine typically increases with the dose until a certain point, where the effect stagnates or decreases—just as the test accuracy in our dimension finding problem. The optimal dose is approached by statistical testing procedures similar to ours (Budde & Bauer, 1989; Bauer & Budde, 1994).

#### Put together: The dimension-testing procedure

Our procedure detects the dimensionality of triplet data by estimating psychophysical scales and looking for their accuracy peak with a sequential testing scheme:

(a) Estimate scales for d=1 to m+1:
  (1) Estimate and cross-validate k psychophysical scales in d dimensions with soft ordinal embedding, repeat r times on shuffled triplets.
  (2) Collect triplet accuracies ${\mathrm{acc}}_{d}\in R^{rk}$ from the r-repeated k-fold cross-validation.
(b) Test scales pairwise for d=1 to m:
  (3) Calculate the $p_{d}$ value of an accuracy gain $H_{d}:{\mathrm{acc}}_{d+1}-{\mathrm{acc}}_{d}>0$ with the Student t-distribution PDF (df=kr-1) at the t-value of Equation 4.
(c) Combine the tests for d=1 to m:
  (4) Accept $H_{d}$ if $p_{d}<\frac{\alpha }{m-R(p_{d})+1}$, R is the rank of $p_{d}$.
  (5) If $H_{d}$ rejected, return “d dimensions.”
(d) If no $H_{d}$ has been rejected, return “at least m+1 dimensions.”

### Simulations: Validating our procedure

New methods should always be validated against ground truth, but ground-truth dimensionalities do not exist in “real” psychophysical data. Hence we simulated data from “synthetic” observers. By simulating judgments, we have complete control over all aspects of the data and thus can rigorously assess our statistical procedure.

#### Generated ground-truth scales

To cover a range of experiments with our simulations, we require ground-truth scales where we can freely choose the number of stimuli or dimensions and recreate comparable variants of the scale. Thus, we sampled the scale’s coordinates from normal distributions, which provides us with an infinite amount of ground-truth scales, called *normal scales* in the following. In addition to these normal scales, results of two ground-truth scales inspired by actual psychophysical scales, namely, a circle-like hue (Ekman, 1954) and a helix-like pitch scale (Shepard, 1965) are available in the Supplementary Material E.

The normal coordinate distribution $\psi_{1},\cdots ,\psi_{n}∼N\left(0,I\right)$ has zero mean and an identity covariance matrix (maximum density at the origin). Reproducibility is assured by seeding the pseudorandom generator used to sample from the distribution. Different numbers of stimuli n and dimensions d were chosen to simulate different psychophysical stimuli: Small experiments of n=20 and d={1,2,3}, medium experiments of n=60 and d={1,2,3,8}, and large experiments of n=100 and d={3,8}.

For all these ground-truth scales and simulated triplets, our procedure searched the true dimensionality from 1 to (d+2), as shown in the following sections.

#### Simulated triplet judgments

As in a laboratory experiment, we created random triplets of stimulus indices. However, instead of asking observers to judge, we calculated judgments from distances in a ground-truth scale plus judgment noise. For each ground-truth scale, we created multiple datasets with a different number of trials.

Comparing validation results from different ground-truth scales requires “a common currency” of the evaluated metrics. However, the quality of scale estimates and, thus, our dimension estimates depend not on the absolute number of trials but on the ground-truth dimension d and stimulus number n. Therefore comparable trial numbers were calculated with the scaling factor λ and the λdnlogn- formula (Haghiri et al., 2020), based on mathematical proofs in the computer science literature. We used the natural logarithm (an arbitrary decision) and varied λ to define three different dataset sizes: The “minimal” dataset (λ=2), the “moderate” dataset (λ=4), and the “generous” dataset (λ=8). In addition, a sample of 10,000 triplets accompanied every triplet dataset to approximate the *noise ceiling*, the best possible generalization accuracy considering the fraction of triplets that became incompatible through the (simulated) judgment noise.

We created the dataset’s triplets by random sampling of three distinct stimulus indices, i,j,k, that were judged from the Euclidean distances between ground-truth positions $\psi_{i},\psi_{j},\psi_{k}$ as

$$\begin{array}{c} \begin{array}{cc} (i,j,k), & \mathrm{if}\;\mathrm{dist}\left(\psi_{j},\psi_{i}\right)+\varepsilon \leq \mathrm{dist}\left(\psi_{i},\psi_{k}\right)\\ (i,k,j), & \mathrm{otherwise}. \end{array} \end{array}$$

At every judgment, the noise component ε was sampled from a normal distribution $N(0,\sigma^{2})$ to simulate judgment noise as in similar simulation studies (Devinck & Knoblauch, 2012; Aguilar et al., 2017; Haghiri et al., 2020). The normal noise models observers that misjudge closely perceived similarities more frequently, that is, visual similarity judgments between three different red apples should be less consistent than two red apples and one green pear. Three noise levels σ were defined as low (σ low =0.5, e.g., controlled laboratory experiments), medium (σ med =1.0), or high judgment noise (σ high =2.0, e.g., online experiment); the interested reader can find a visualization of these levels in the Supplementary Material C. We rescaled these noise levels according to distance’s spread to maintain comparable signal-to-noise ratios across different simulation settings and approximately match the triplet accuracy range in corresponding human datasets.

#### Accuracy peaks at the ground-truth dimension

The first results we look at are accuracy-by-dimension graphs as the underlying metric of our procedure, whose key idea is to identify a test accuracy peak at the ground-truth dimensionality. The following representative results use datasets with the three-dimensional-normal scale (n=60). Datasets with varied dimensionality and number of stimuli but comparable results are shown in Supplementary Material G.

**Figure 2.**
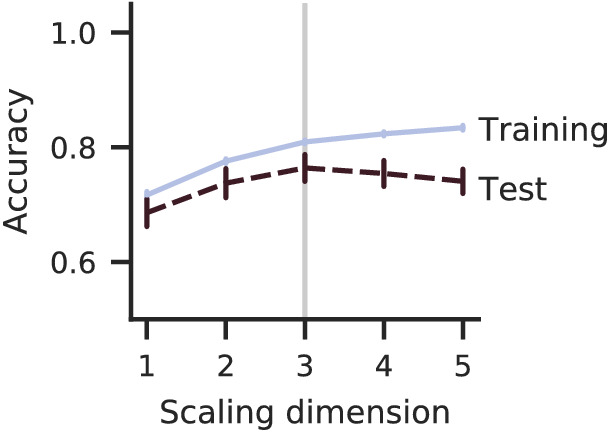
Comparison of training and test triplet accuracies for different embedding dimensionalities (#triplets=2947,λ=4, noise = med ). The triplets are simulated with medium judgment noise from an artificial three-dimensional scale with 60 normally distributed points. The training accuracy increases with the dimensionality, but test accuracy peaks at the ground-truth dimensionality (vertical line). The standard deviation between cross-validation folds (error bars) is higher for the test accuracy.

The accuracy on training triplets in Figure 2 increases with the embedding dimensionality as the scale fits more and more triplets. However, the accuracy on test triplets peaks at 3D, the ground-truth dimensionality indicated by the vertical line, and shows that the scale is overfitting to noisy triplets for higher dimensionalities.

**Figure 3.**
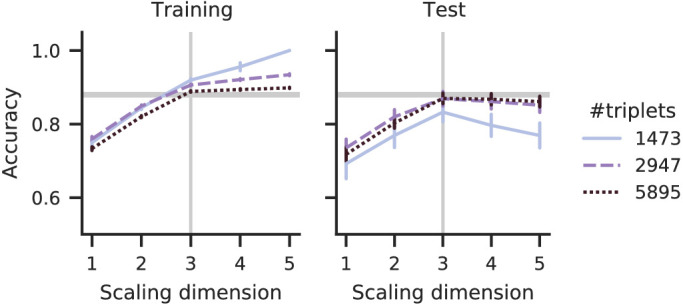
Accuracies for different dataset sizes and low simulated noise. Dataset sizes affect the test accuracy peak only mildly; it has a stronger influence on how much accuracy increases (train; left panel) or decreases (test; right panel) after the ground-truth dimensionality (vertical line).

The effect of dataset size on the test accuracy is negligible for scales of ground-truth dimensionality but not for scales with higher dimensionalities: Figure 3 (right) shows a pronounced accuracy peak for the small dataset, but the accuracy for larger datasets converges to the noise ceiling (horizontal line). The dataset size also influences the slope of the training accuracy, such that the training accuracy of a large dataset just minorly increases beyond the ground-truth dimensionality. This reduced slope of training and test accuracies reduces their difference by increasing the dataset size.

**Figure 4.**
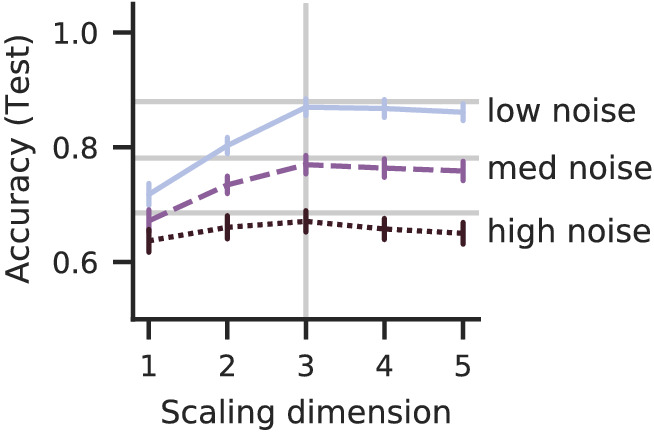
Test accuracies for simulated noise with different signal-to-noise ratios (# triplets =5895,λ=8). The noise reduces the best-possible accuracy (noise ceiling, horizontal lines) leads to flat accuracy graphs. The high noise accuracy shows no peak at the ground-truth dimensionality (vertical line).

In contrast with dataset size, noise severely reduces the noise ceiling and thus the achievable accuracies (Figure 4). Additionally, noise flattens the accuracy graph, which thus shows less pronounced peaks, which might decrease the precision of dimension estimates.

#### Estimated dimensionality is conservative

The following results are our procedure’s dimensionality predictions, based on statistical tests for a gain in accuracy.

**Figure 5.**
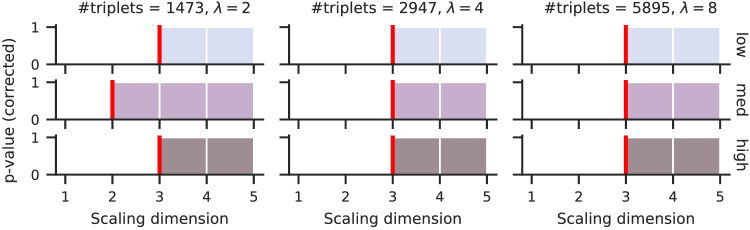
The p-values of statistical tests to detect accuracy gains by adding a dimension to the estimated scale from simulated triplets of a three-dimensional ground-truth scale with 60 stimuli. Colors and vertical order match the noise levels of Figure 4. The predicted scale dimensionality (red lines) matches the three-dimensional ground-truth in most settings; for more than three-dimensional, the accuracy gain was always rejected (p>.05).

The statistical test’s p-values below α=.05 indicates a significant gain in accuracy by adding another dimension to the scale. Figure 5 shows these p-values along with the predicted dimensionality at the first rejection of the gain hypothesis (red line) for multiple noise levels and dataset sizes. The accuracy peaks were reliably detected at the ground-truth dimensionality even for settings where the peak is barely visible (compare high noise graph in Figure 4). Only for one small dataset was dimensionality underestimated (left panel middle row in Figure 5).

Across all 81 simulations of normally distributed ground-truth scales, our procedure estimates the correct dimensionality 73% of the time. All incorrect predictions underestimated the ground-truth dimensionality. These underestimates occurred more frequently for small datasets, high noise, or large ground-truth dimensionality. The individual dimensionality predictions are summarized in Supplementary Materials F. Please note that 73% correct might appear low; however, this is only a reflection of the fact that we used very challenging simulation conditions with (sometimes) just the minimal amount of data and substantial noise. Our results clearly show the considerable influence of noise on correct dimensionality estimation. If we only consider low-noise settings, 93% dimensionalities (25 of 27) were predicted correctly, even including the small datasets. The incorrect predictions were with datasets of few stimuli given the ground-truth dimensionality (three-dimensional and n=20; eight-dimensional and n=60). This result indicates that the stimulus number might be another factor affecting the robustness of dimensionality estimation; this factor is common to all dimension estimators and is addressed in the final Discussion.

#### Repeated simulations show reproducability

In the previous sections, we showed single runs of our method on various datasets to investigate the relevant parameters. Here, we repeat the procedure 100 times on the same dataset to evaluate the robustness of the procedure. This section shows results for triplets from the eight-dimensional normal scale (n=100), and comparable results on different datasets are available in the Supplementary Material G.

**Figure 6.**
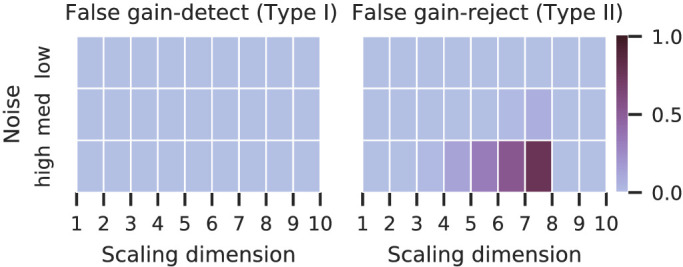
Unexpected rejections and detections of our neighboring-dimension tests for repeated simulations of eight-dimensional normal scales (n=100) with medium triplet size (λ=4). With noisy triplets, the accuracy gain is rejected, even before the ground-truth dimension leading to lower-bound estimates of the dimension.

The procedure consists of statistical tests to detect if adding a dimension increases the accuracy (compare *p*-values in Figure 5). We expect significant increases until the dimensionality equals the ground-truth scale’s dimensionality. Figure 6 depicts how many of our procedure repetitions violate this expectation. The left plot shows that almost no incorrect accuracy gain was detected, which is expected from the conservative multiple-testing correction and the robust decrease of test accuracy after the ground-truth dimensionality in the previous plots. The dark colors in the right plot indicate that the statistical test rejected the accuracy gain hypothesis for some scaling dimensions lower than the ground truth; the procedure underestimated the dimensionality. These underestimates occur more frequently for high noise settings (Figure 6, right) and for small datasets and high ground-truth dimensionality (see Supplementary Materials F). Overall, the repeated runs of our method confirm the large influence of the noise magnitude on scaling accuracy and dimensionality underestimation.

#### Summary

Our procedure reliably identifies the ground-truth dimensionality in the simulated datasets if enough trials were collected and the noise is low. Collecting more trials can only partially offset the noise; thus, the focus should be on controlling judgment noise through control measures in the experiment. However, our procedure identifies a lower-bound dimension estimate even in the worst-case conditions of high noise and few trials. This dimensionality underestimation of sparse and noisy data is—in our opinion—preferable behavior because it provides the user with a more straightforward explanation. Such worst-case conditions are identified easily by monitoring the train and test accuracies. A large gap between training and test accuracy and the considerable variation of accuracies within cross-validation folds indicates that more trials should be collected, whereas low accuracy indicates considerable noise.

## Dimensionality of human data

The results presented in the previous section showed that our procedure could predict the dimensionality of *simulated trials*. However, the intended application of our procedure is dimension estimation on *behavioral trials* from psychophysical experiments. Thus, we also investigated behavioral datasets. In contrast with simulations, behavioral data have no ground truth, but just more or less evidence about the “correct” dimensionality.

The section starts with hue triplets as a sanity check, where we expect and find a two-dimensional representation and continues with two other datasets where the true dimensionality is less evident. Its prediction is—perhaps—somewhat surprising.

### Hue: Verified expectations

Color is a natural testbed of multidimensional perceptual spaces. One property of colors, the hue, might be represented with a color wheel that requires two euclidean dimensions, even though the corresponding physical parameter is one-dimensional (wavelength of light). The hue similarity is typically collected in rating experiments; however, we computed triplet trials from the ratings.

The ratings of 36 different hues were collected by Bosten and Boehm (2014) from 18 observers. Every hue was presented in 3 trials such that every observer answered 108 trials. On each trial, a test patch was presented, and the observer rated (from 0 to 9) the similarity of the patch’s hue to red, yellow, green, and blue; thus, the observer supplied four numbers on each trial. For example, observers experience a violet hue with red and blue but with little yellow and green. One might think of these ratings as samples in a four-dimensional space with a red, yellow, green, and blue axis that can be used to judge hue triplets. Per triplet, we randomly selected a target and two other hues and calculated the Euclidean distance of the corresponding four-dimensional ratings to judge which hues were more similar. This way, the triplets involve—to a certain degree—the behavioral noise in contrast to the simulated noise in the previous section.

**Figure 7.**
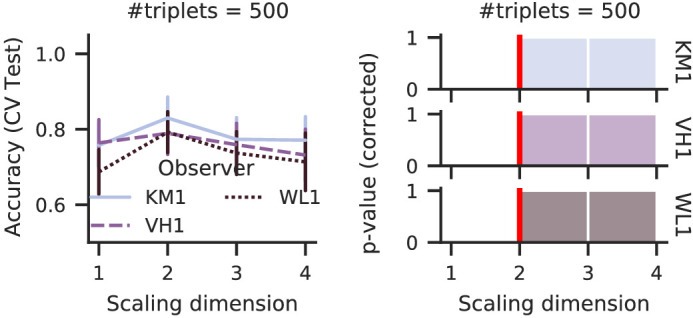
The clear estimation of two perceived hue dimensions matches the color wheel representation even if the original data were the four-dimensional ratings of (Bosten & Boehm, 2014).

Figure 7 shows our procedure’s estimates for hue triplets of three arbitrarily picked observers. For all of them, our procedure suggested a two-dimensional scale that fits very well with the assumed color-wheel representation of the hue. We note that neither data collection nor triplet sampling involved a two-dimensional bias; instead, the data were collected as four-dimensional ratings.

### Slant from texture: Revealed influences

Another common, but less apparent, percept of interest is the slant of angled textured planes (Rosas, Wichmann, & Wagemans, 2004; Rosas, Wagemans, Ernst, & Wichmann, 2005; Rosas, Wichmann, & Wagemans, 2007). The common assumption is that slant and angle are single-dimensional and relate monotonically.

Here we used a triplet-dataset of slant-stimuli by (Aguilar et al., 2017), where observers compared three dot textured planes (“polka dots” by Rosas et al., 2004) per trial, which varied in eight angles. In total 840 triplets were collected, such that each triplet shows angles (left < anchor < right). The ordering is a restriction of the MLDS algorithm (Knoblauch & Maloney, 2012) that was used to estimate the scales in the original publication (Aguilar et al., 2017). Using MLDS, they could only consider one-dimensional, monotonic scales. Following up, Haghiri et al. (2020) reanalyzed these triplet data with ordinal embedding algorithms to relax the monotonicity assumption and observed a surprising “dip” of slant.

**Figure 8.**
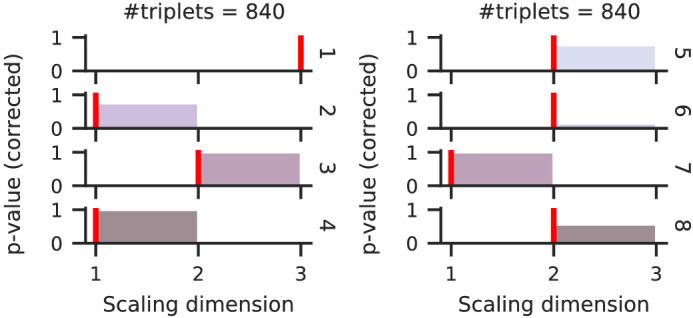
The optimal scaling dimension of slant varies between eight observers (rows), according to our procedure—just three observer’s scales were one-dimensional as expected. *p*-values below α=.05 indicate rejection of the $H_{0}$ = “No accuracy gain by adding a dimension.”

Here, we even further question the assumption that the resulting scale has to be one-dimensional by applying our procedure. Perhaps surprisingly, our procedure predicts multidimensional scales for some observers: The one-dimensional scale was suggested for three out of eight observers (Figure 8); the other scales were estimated as two (four observers) or even three-dimensional (one observer).

A two- or three-dimensional slant scale is contraintuitive given that only the angle varied in the experiment. As there is no ground-truth dimensionality, this surprising result may question our procedure’s reliability or indicate that additional dimensions were observed. The reliability of our procedure was thoroughly tested in simulation experiments, and in no condition—not once—did our procedure overestimate the dimensionality; even in deliberately poor datasets, the dimensionality was underestimated, so there is no reason to believe that our procedure failed for this slant dataset.

The alternative explanation, additional perceived dimensions, could be related to the stimulus design. Even though the independent slant variable is one-dimensional, the stimulus is a high-dimensional image of a dot pattern. Observers had no direct access to the slant angle. However, they must have inferred it from one or several stimulus properties, such as changes in the size, width, aspect ratio, or density of the texture elements. Perhaps some of the observers switched between the cues they used or changed their cue combination strategy as a function of the angle. Clearly, without further experiments, this issue cannot be settled. However, to us, it indicates that one should always consider multidimensional scales if only to confirm that a presumed one-dimensional relationship is indeed one-dimensional.

### Eidolon’s distortions: Correlated parameters

**Figure 9.**
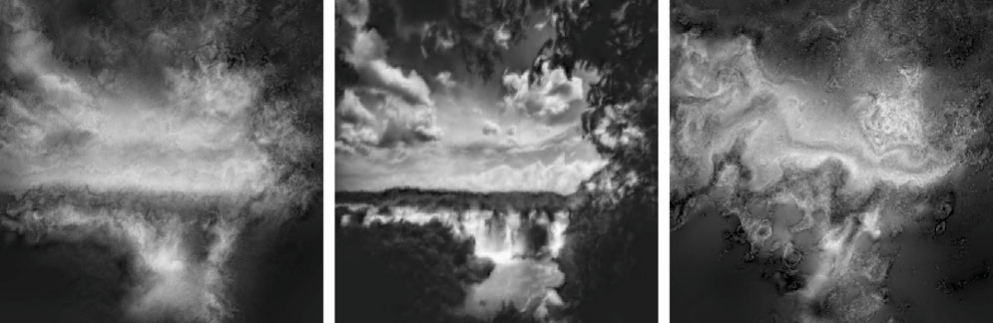
Three distortions of the same landscape image, created with the Eidolon factory and used as stimuli in the laboratory experiment of (Haghiri et al., 2019).

The third dataset uses high dimensional stimuli, as shown in Figure 9, distorted versions of landscape photography that differ in most pixels. However, the distortions are defined by three parameters *reach, grain*, and *coherence* of the Eidolon factory (Koenderink et al., 2017). Triplets of 100 such distorted stimuli of the same landscape photography were generated by (Haghiri, Rubisch, Geirhos, Wichmann, & von Luxburg, 2019) for their laboratory experiment. Their observers were asked 6,000 random triplet questions and responded to almost all of them (first observer, 6,000 responses; second, 5,996; third, 5,999).

**Figure 10.**
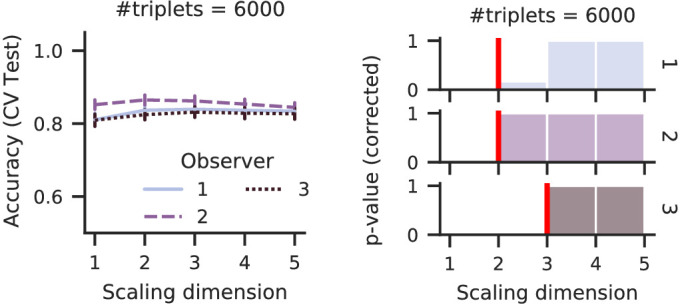
The optimal scale for the eidolon triplets is two-dimensional, which supports the observations of Haghiri et al. (2020). This result counters the first intuition of a three-dimensional scale because the stimuli were created with a three-dimensional distortion algorithm.

From the three parameters of the Eidolon factory, one might expect three perceived dimensions, but previously (Haghiri et al., 2020) observed a peak in mean accuracy for two-dimensional scales. Our procedure also predicts a two-dimensional scale for two observers and a three-dimensional scale for one observer (Figure 10). Again, from our simulations, we believe we are unlikely to overestimate the perceptual dimensionality. Furthermore, we observe relatively high accuracies and only a small gap between train- and test accuracy, indicating that the noise in the dataset is relatively low (and, thus, our dimension estimates are very likely correct).

Observing two perceptual dimensions given the three perturbation parameters of Eidolon means that multiple image generation parameters lead to similar percepts, that is, at least two observers did not perceive the (subtle) differences between all the perturbations.

## Discussion

We propose a procedure to estimate the appropriate dimensionality in psychophysical scaling. Our procedure is based on model selection and statistical hypothesis testing to provide a more objective decision than previous approaches. We show in simulation studies that this procedure can recover the ground-truth dimension and produces conservative estimates in noisy settings where “classical” dimension estimators typically overestimate the dimensionalities.

Using three existing behavioral datasets, we showed the use of our procedure in practice; in the case of color, we confirmed the expected two-dimensional embedding: the hue or color circle. For the slant-from-texture and eidolon experiments; however, our procedure uncovered higher (slant from texture) or lower (eidolon) embedding dimensions than one might have predicted based on the number of explicitly manipulated variables in the experiments (one and three, respectively).

### Robust perceptual dimensionality estimation

The robustness of our procedure’s predictions was validated in multiple simulation experiments. These validations are essential because one can not compare with ground-truth, and errors in the procedure would be taken for reality. However, the validity of simulation-based validations is based on assumptions that link the simulations with the behavioral studies to which our procedure should be applied. In psychophysical scaling, we expect few observers to judge many trials in a well-controlled laboratory environment. Observers are analyzed separately, so responses are consistent, low-dimensional, and of low perceptual noise (Gaussian distributed).

If an experiment is of this type, we have shown the reliability of our procedure and are confident that it returns accurate scale and dimension estimates.

### Lower bound estimates from ill-defined data

Typical psychophysical datasets are known for their high level of control. Yet, circumstances—for example, large-scale online experiments with little to no control over the screen, room, attention, noise levels, and so on—can lead to too few trials or too much noise, that is, too many random responses. These data deficits decrease the scale’s accuracy. Ultimately low-quality data lack the information required to reconstruct the original scale. Our simulations showed that large noise in the data is the most detrimental factor: Doubling the noise cannot be compensated for by doubling the amount of data. In low data quality scenarios—large noise—our simulations show our procedure to err on the conservative side, that is, to propose a lower dimensional scale than ground truth. Again, we believe this to be a feature rather than a bug in the context of inferring perceptual dimensions.

Recognizing whether a dimension estimate is lower than expected because the perceptual space is low dimensional or because the dataset is too small and noisy is obviously essential. Two metrics need to be inspected to decide between these two possibilities: First, the maximum test accuracy and, second, the difference between training and test accuracy. Low-noise settings show a maximum accuracy of about 0.9; accuracies of less than 0.7 are critical and indicate high noise, and thus an increased risk of dimensionality underestimates. Estimates derived from small datasets show low accuracies, too, but are easier to detect by comparing the number of triplets with, e.g. the 2dnlogn-rule (Haghiri et al., 2020), for different hypothesized dimensionalities d. Additionally, a large difference between train and test accuracy (≫0.1) can also indicate a lack of data.

The lack of data can be resolved by running additional lab sessions, but reducing the noise might be more difficult. Typical strategies to reduce the noise involve a well-controlled lab environment (Wichmann & Jäkel, 2018; Haghiri et al., 2019), varying the task and extending training sessions (e.g., triplets instead of Likert ratings; Demiralp, Bernstein, & Heer, 2014) or post hoc data cleaning (e.g., dropping blocks where repeated trials disagree; Lagunas et al., 2019).

### Estimates of high-dimensional spaces

In recent years, there is a trend to investigate perceptual space in large scale online experiments using stimuli like object photographs (Roads & Love, 2021; Hebart et al., 2020). In these studies, the data of very many observers are pooled and then jointly embedded.

The perceptual spaces identified in the above studies tend to be rather high dimensional. However, this high dimensionality might not necessarily reflect the “the internal human object perception space,” but might instead be (partially) an overlapping *super-space* (Carroll & Chang, 1970), composed of the multiple observer’s (cognitive) decision criteria.

This possibility of obtaining compositional super-sets from such experiments makes it difficult to reconstruct individual perceptual spaces from representational accuracy. It is, thus, not an intended application of our procedure.

Furthermore, we would like to highlight the general difficulty of estimating dimensions if their value is high. Intuitively, a space is d-dimensional if its points cover a (small) cube of d dimensions. However, the number of points that is needed to cover a d-dimensional cube grows exponentially with the dimension d. To see this, imagine 10 data points that cover the one-dimensional interval [0,1], for example, the grid points 0.1,⋯,0.9,1. To cover a two-dimensional cube similarly well, we would already need 10×10=100 data points. In general, to cover a D-dimensional cube we would need on the order $10^{d}$ many points. This fact makes it very difficult to estimate the dimension from a sample of points when D is large. It is pretty much impossible to have enough sample points to be able to distinguish between, say, a space of 50 versus a space of 51 dimensions: our sample points will neither cover a cube of 50 nor of 51 dimensions, making each such estimate (or corresponding test) utterly unreliable. A more formal argument for the difficulty of estimating high dimensions can be found in Block, Jia, Polyanskiy, & Rakhlin (2021). Consequently, although it is well possible in psychophysics to discriminate a two-dimensional from a three-dimensional space, it seems pretty much impossible to discriminate between, say, 50-dimensional versus 51-dimensional or 50-dimensional versus 60-dimensional. Even in a setting with very low noise, the high-dimensional scenario would require a prohibitively large number of data points (stimuli) and triplet trials for dimensionality estimation. In psychophysics, it might often be better to avoid high-dimensional spaces from the outset by using well-designed stimuli that observers judge by a few criteria.

## Supplementary Material

Supplement 1
