# Supplementary material for: Estimating the perceived dimension of psychophysical stimuli using triplet accuracy and hypothesis testing
Source: J Vis. 2022 Dec 5;22(13):5. doi: 10.1167/jov.22.13.5 (PMC9730733; doi:10.1167/jov.22.13.5)
Supplement: Supplement 1 [file jovi-22-13-5_s001.pdf]

Supplementary Materials

A Variations of similarity judgment tasks

All experimental tasks used for psychophysical scaling measure the relative similarity between stimulus pairs, either directly as in rating experiments or indirectly as in JND-based or comparison-based methods used in the work at hand (see main paper). Experiments with comparison-based methods ask observers to order the perceived similarity or dissimilarity of stimulus pairs in one way or another. The ordering is most apparent in the quadruplet task, which asks per trial which of two pairs is most similar (dissimilar), and in the triplet task, which asks for three stimuli if left or right is more similar (dissimilar) to the centre. The triplet task can be generalized to n-choose-k and n-rank-k tasks presenting n stimuli in addition to the anchor and asking for the k of n most similar (dissimilar) stimuli and in n-rank-k tasks for their ranking. A response to these tasks can be used to calculate multiple triplets of the anchor, the chosen, and another presented stimulus. Different tasks without anchor stimuli ask for the odd-one-out or the most central of three presented stimuli. The pairwise comparisons are most dissimilar for the odd-one-out and most similar for the most-central stimulus.

Table 1 summarises possible conversions from various comparison-based tasks to triplets, such that these responses are usable with our proposed procedure. These converted triplets are partially dependent such that scaling performance might not be comparable between sampled and converted numbers of triplets. From a psychological perspective, one should be even cautious when comparing responses from conversions, because the actual task, the instructions, and the context (i.e. fewer or more presented stimuli) probably influence the responses. Mathematically these conversions are sound as long as the triangle inequality holds in the perceptual space, a reasonable assumption.

Table 1. Conversions from other comparison-based tasks to triplets. Triplets denote the response by order of the stimulus indices (anchor, chosen, other), and curly brackets are a short-notation for repetition of the same triplet with all index variants in the bracket, e.g. $(1, \{2, 3\}, \{4, 5\})$ means triplets $(1, 2, 4), (1, 2, 5), (1, 3, 4)$, and $(1, 3, 5)$.
In the examples below, we denote duplicated triplets with exchanges in one position by curly braces.

Task	Presented	Chosen	Triplet mapping	Example of task
8-choose-2	$(i, j, \ldots, q)$	j, k	$(i, \{j, k\}, \{l, \ldots, q\})$	Roads and Mozer (2019)
8-rank-2	$(i, j, \ldots, q)$	j, k	prev. and (i, j, k)	Roads and Mozer (2019)
odd-one-out	(i, j, k)	k	(i, j, k) and (j, i, k)	Hebart et al. (2020)
quadruplet	$((i, j), (k, l))$	(i, j)	$(i, j, l$ or $k)$ if $i == k$ or l<br>$(j, i, l$ or $k)$ if $j == k$ or l	Maloney and Yang (2003)

B Normal distribution of accuracy samples

The procedure proposed in this work assumes normally distributed test accuracies from repeated scale estimates, which is grounded in this paper's main part from a theoretical perspective. Here we show the practical grounding in the form of a brief simulation experiment and a statistical test for normality.

We looked at two different accuracy samples: Accuracies from 100 independently simulated triplet datasets of the same ground-truth scale to approximate the actual accuracy distribution, the so called *hand-off* accuracies, and cross-validation

accuracies of a single simulated dataset such as is used in our procedure. We simulated every dataset of $2,000$ triplets with a 3D-normal scale (medium noise) as described in the paper's simulation section. In the hand-off setting, we estimated the scale with $1,800$ triplets and calculated the accuracy with 200, and in the cross-validation configuration, we used ten repetitions of 10-folds.

The histogram of both accuracy samples is shown in Figure 11 along with the sample means (vertical line) and intervals of two standard deviations (dashed line). The hand-off accuracy means, used as a proxy of the actual accuracy, is included in the corrected interval of the cross-validation samples but overestimates the accuracy spread. The normality of both accuracy samples was tested with a combined skew and kurtosis test (D'Agostino & Pearson, 1973). Both settings failed to reject the null hypothesis; their samples are normally distributed (CV: $s^2 + k^2 = 2.46, p > .05$, test set: $s^2 + k^2 = 0.47, p > .05$).

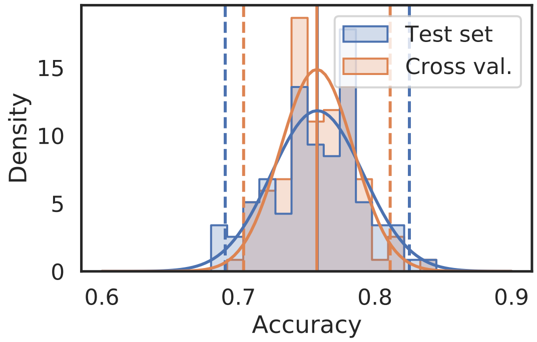

Figure 11. Histogram of accuracy samples from independent test sets and cross-validation. Vertical lines show the mean and two standard deviations (dashed), and the line shows a corresponding normal distribution PDF. The cross-validation samples have a smaller deviation, which is the variation underestimated to be corrected in statistical tests (Nadeau & Bengio, 2003).

C Noise visualization

The influence of triplet number and the judgment noise on scale estimates is illustrated in Figure 12 by showing scale estimates from multiple noisy simulation runs. The coordinates were aligned in terms of rotation, scale, and translation with the Procrustes method (Gower, 1975).

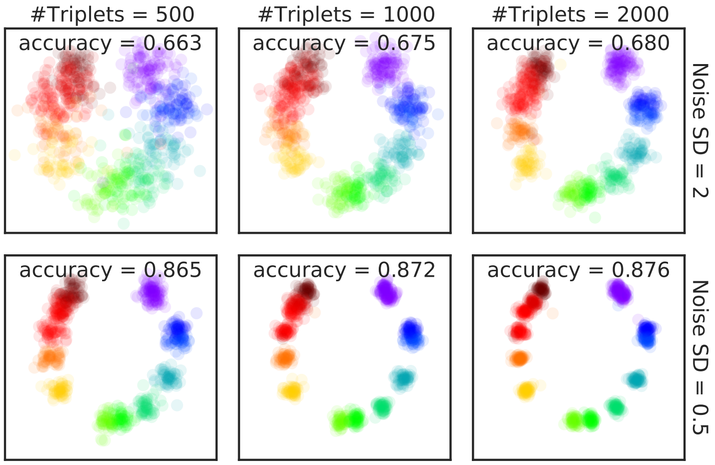

Figure 12. The robustness of hue embeddings increases with the triplet number and decreases with the judgment noise.

D Algorithm details

The pseudo-code Algorithm 1 shows the algorithmic details of repeated cross-validation and the testing corrections used in our dimension testing procedure.

Algorithm 1 Procedures for repeated CV and corrected t-test.

function REPEATED_CV_SCORES(EMBEDDING, SCORE, $\mathcal{T}, r, k$)
  for all $j \in 1..r$ **do** ▷ Repeat CV r times.
    $\mathcal{T}^* \leftarrow$ SHUFFLE$(\mathcal{T})$
    $\mathcal{S} \leftarrow$ CROSS_VAL_PARTITION$(|\mathcal{T}|, k)$
    for all $(\boldsymbol{s}_{\text{train}}, \boldsymbol{s}_{\text{test}}) \in \mathcal{S}$ **do** ▷ k-fold Cross Validation.
      $X \leftarrow$ EMBEDDING$(d, \mathcal{T}^*[\boldsymbol{s}_{\text{train}}])$
      $\boldsymbol{u}_{i,j} \leftarrow$ SCORE$(X, \mathcal{T}^*[\boldsymbol{s}_{\text{test}}])$ ▷ Evaluate on test triplets.
    end for
  end for
  return $\boldsymbol{u}$ ▷ $r \cdot k$ test scores.
end function

function CORRECTED_T_TEST$(\boldsymbol{d}, n_{\text{train}}, n_{\text{train}})$
  $\sigma_{\bar{d}} \leftarrow \sqrt{\frac{1}{|\boldsymbol{d}|} + \frac{n_{\text{test}}}{n_{\text{train}}}} \cdot \text{STD}(\boldsymbol{d})$ ▷ Correction of Nadeau and Bengio (2003).
  $t \leftarrow \frac{\text{MEAN}(\boldsymbol{d})}{\sigma_{\bar{d}}}$ ▷ t-test statistic.
  return STUDENT_T_PDF$(t, \mathit{df} \leftarrow |\boldsymbol{d}| - 1)$
end function

function HOLM_MULTITEST_CORRECTION$(p_1, \ldots, p_k)$ ▷ (Holm, 1979).
  $V \leftarrow \{1, \ldots, k\}$
  for $i \leftarrow k$ to 1 **do**
    $j \leftarrow \arg\min_{d \in V} p_d$
    $p_d^* \leftarrow \frac{p_d}{i}$
    $V \leftarrow V \setminus \{d\}$
  end for
  return $p_1^*, \ldots, p_k^*$
end function

E Simulating a psychophysical experiment

Here we show simulation result where the ground-truth scale is inspired by actual psychophysical scales— the idealized hue and pitch perception as a wheel and a helix.

E.1 The hue perception wheel

This experiment used a two-dimensional hue circle as a realistic example of multi-dimensional ground-truth scales (Figure 1). While we simulate triplets from this ground-truth scale, the ground-truth scale is not artificial but estimated from psychological data. The scale was estimated from pairwise hue dissimilarities (Ekman, 1954) with the multi-dimensional scaling algorithm (Shepard, 1962).

Our procedure correctly estimated two dimensions in most settings, as shown in Figure 13. Just two high noise simulations underestimated the dimension, consistent with the other simulation experiments.

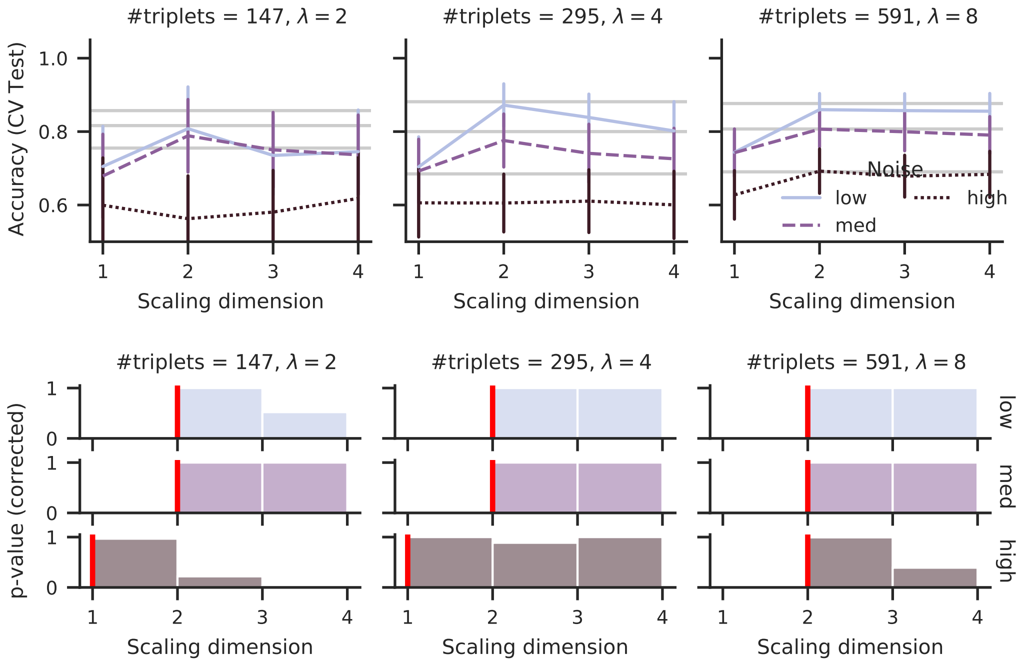

Figure 13. Triplet accuracy and p-values for embedding triplets of a colour wheel to a different dimension. The intrinsic dimension of two is correctly identified (vertical coloured line).

E.2 The pitch perception helix

The ground-truth scale used in this section is a three-dimensional helix (Figure 14), that is inspired from models of pitch perception (Shepard, 1965) but not based on behavioural data. As in the simulation experiments, we created a ground-truth scale and simulated responses, including normally distributed judgment noise. The ground-truth helix has three rotations with 12 tones (an octave) each, where the height of a rotation equals the helix's diameter.

Our procedure reconstructs the three-dimensional structure in the setting with high noise and low accuracy (Figure 15). Surprisingly, the noisier setting shows another reasonably accurate representation with a single dimension, an unrolled version of the helix. This more straightforward, unrolled representation is preferred if less data is available. This trade-off between unrolled 1D and helix 3D representation should depend on the helix's diameter-to-height ratio.

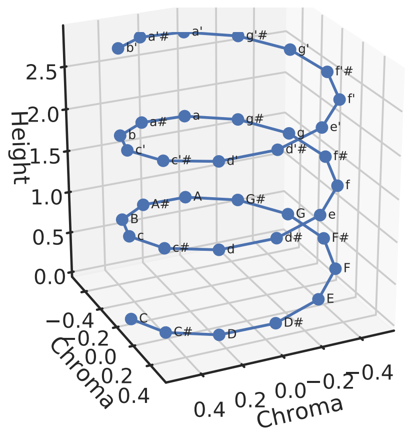

Figure 14. Pitch helix

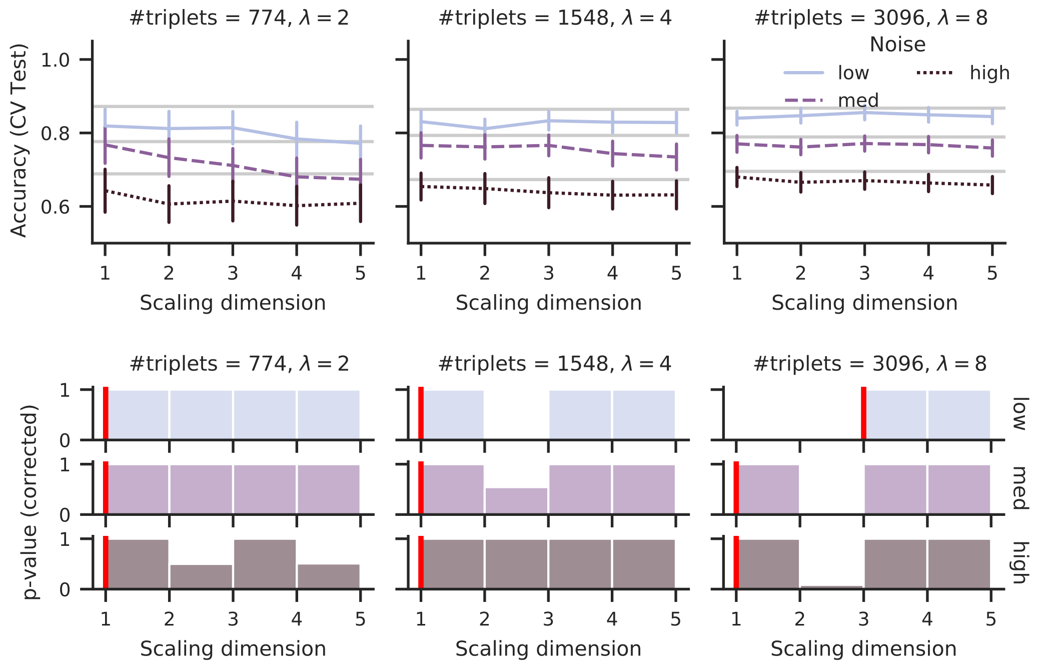

Figure 15. Triplet accuracy and p-values for reconstructing a simulated pitch helix with different judgment noise (colours) and dataset size. The p-values tell two different interpretations: From noisy or small datasets, just the one-dimensional perceived pitch is reconstructed. p-values for the large dataset show, that–provided enough data–a three dimensional representation can represent additional nuances (the helix-like similarities between octaves).

F Overview of simulation results

The Figure 16 shows an overview of dimension estimates across all the *normal* datasets. No simulation overestimated the ground-truth dimension. While most simulations predicted the correct dimensionality, some underestimated it, especially at high noise and large ground-truth dimensions. The noise might shadow distinctive distance information of additional dimensions, so we can interpret these dimension estimates as a lower-bound dimension estimate. In psychological practice, such conservative or lower-bound dimension estimates are beneficial as they provide the simplest model that explains the collected data—given the inherent noise in the data.

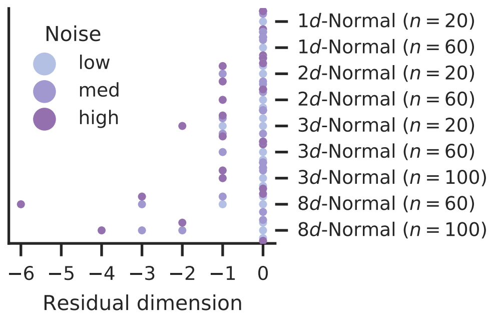

Figure 16. Overview of difference between estimated and ground-truth dimension. Overestimating dimension occurred just for one-dimensional datasets, while underestimation occurred more often for higher dimensions and larger noise.

G Detailed simulation results

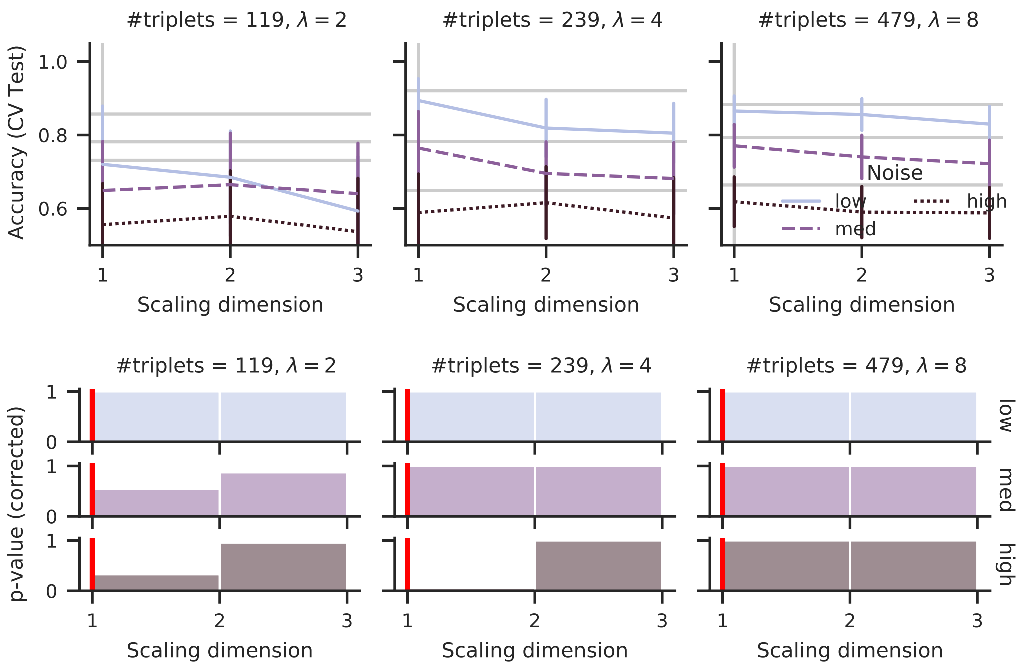

Figure 17. $1D$-normal $(n = 20)$

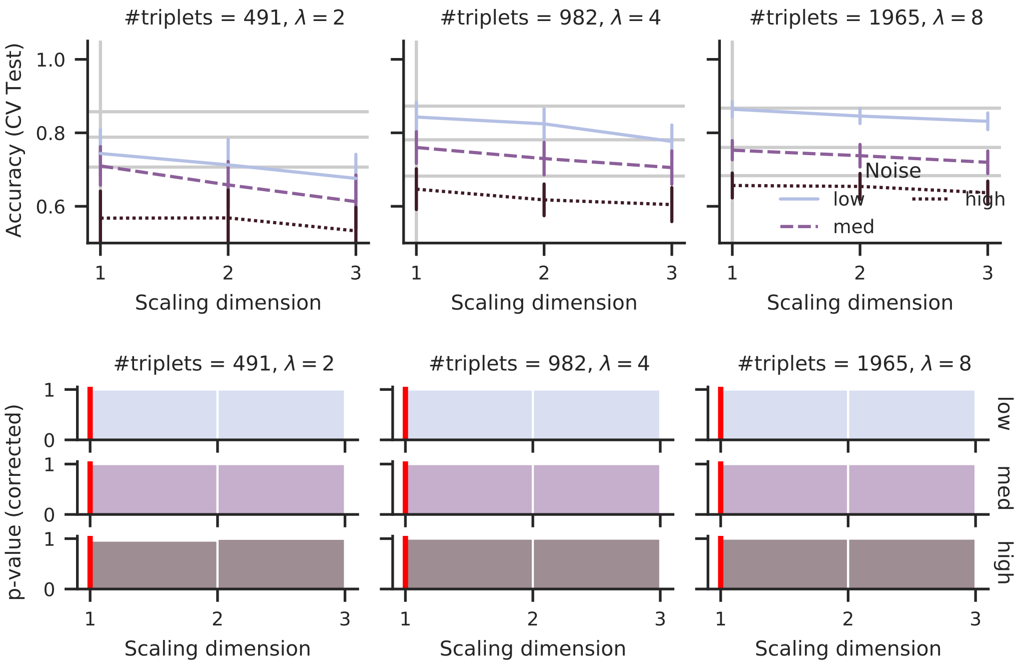

Figure 18. 1D-normal ($n = 60$)

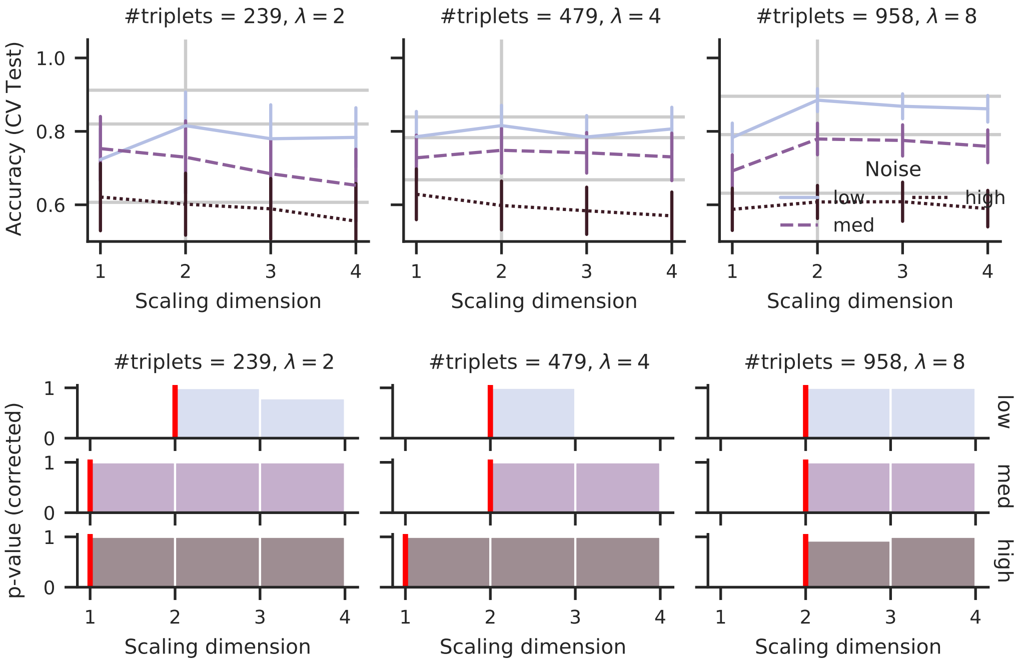

Figure 19. 2D-normal ($n = 20$)

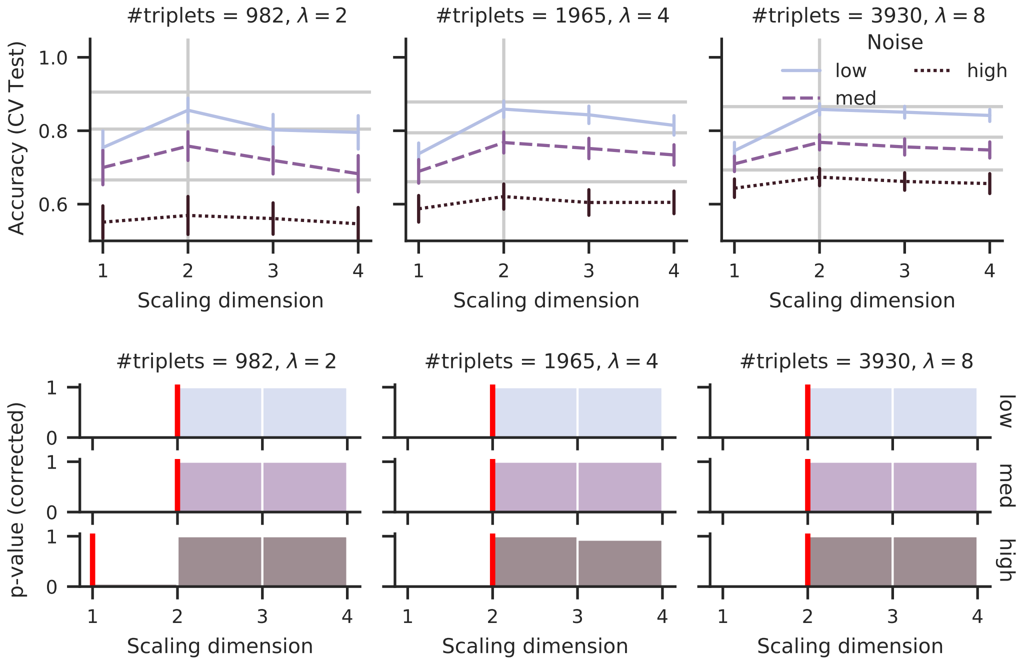

Figure 20. 2D-normal ($n = 60$)

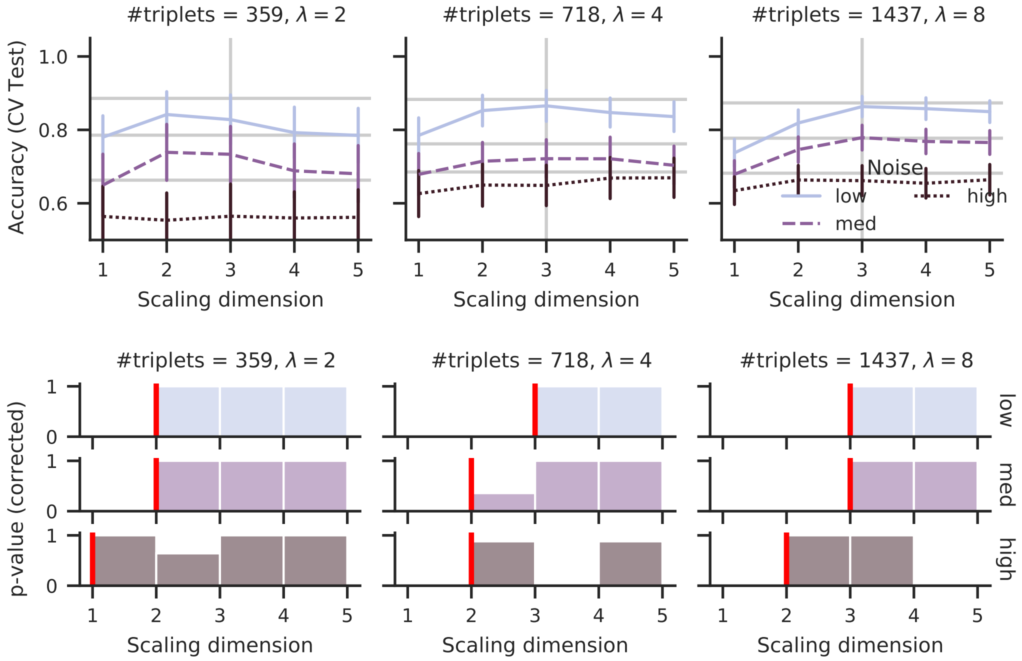

Figure 21. 3D-normal ($n = 20$).

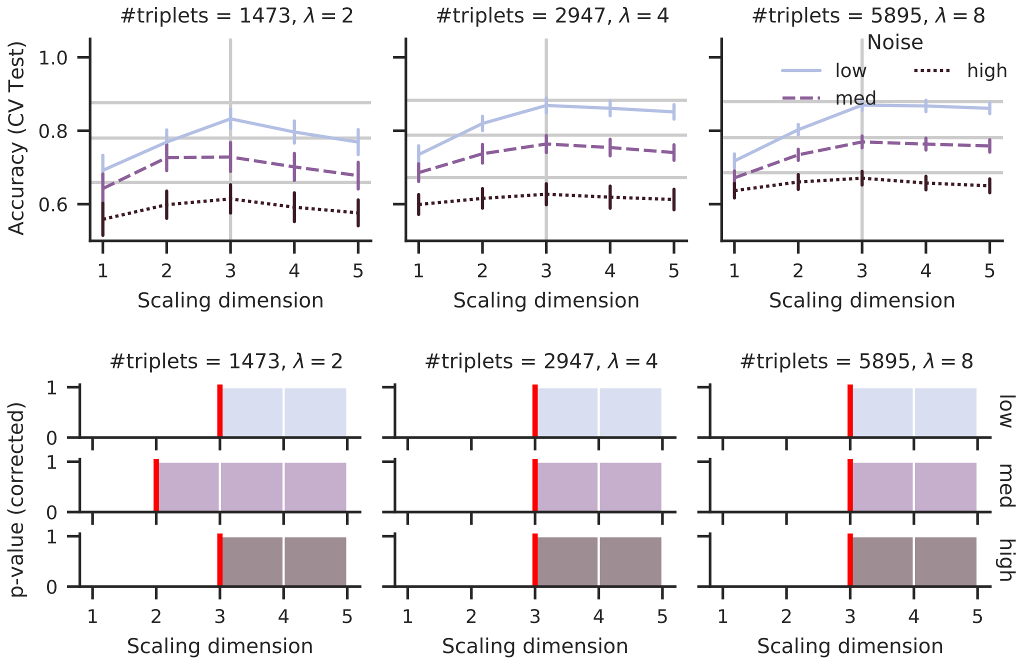

Figure 22. 3D-normal $(n = 60)$

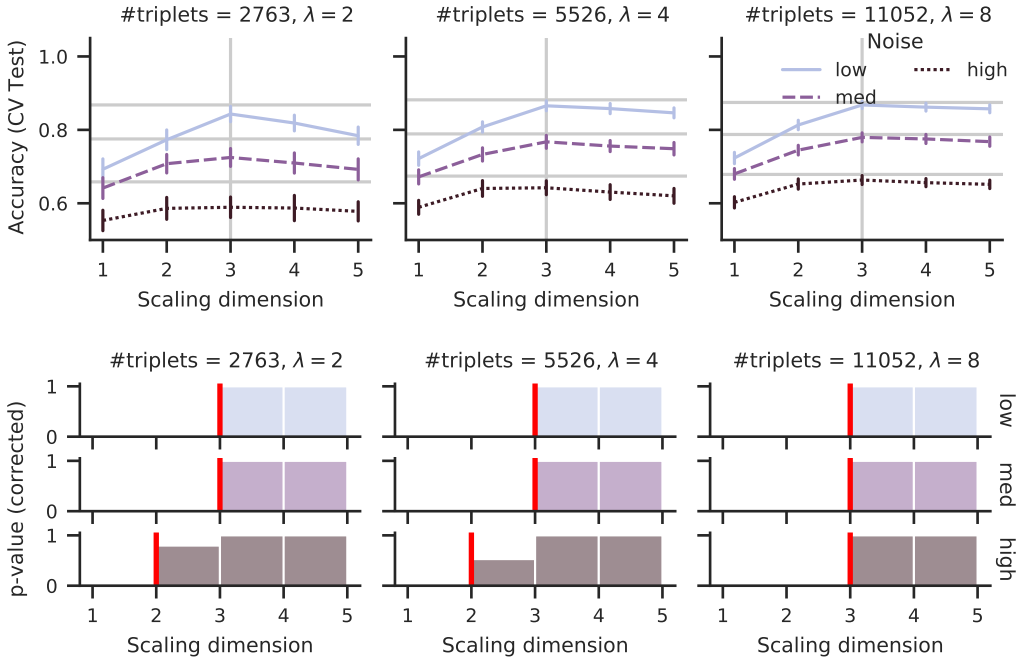

Figure 23. 3D-normal $(n = 100)$

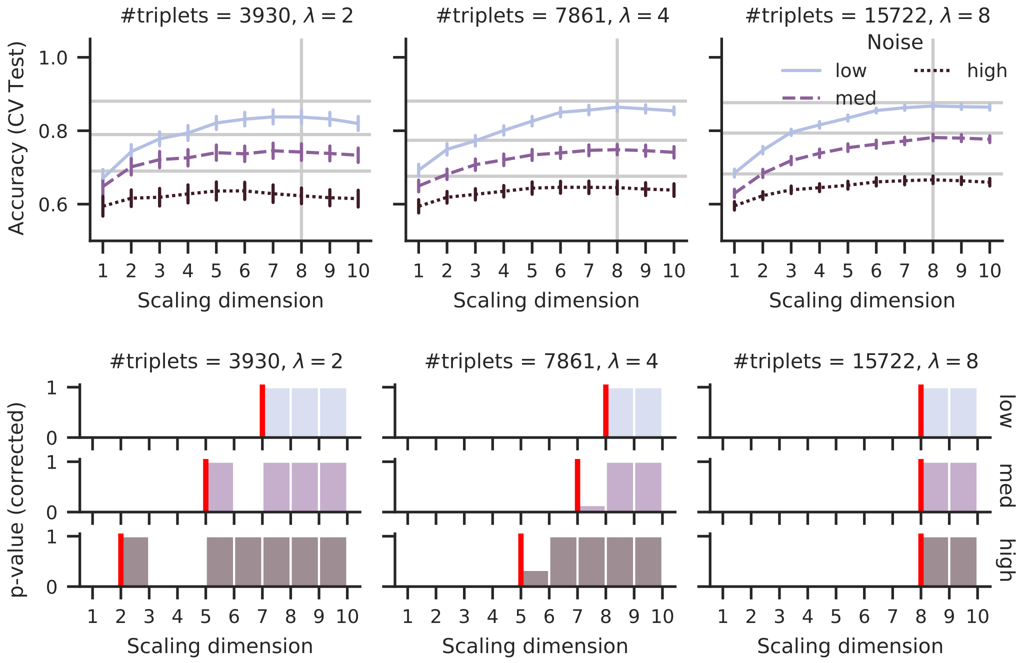

Figure 24. 8D-normal ($n = 60$)

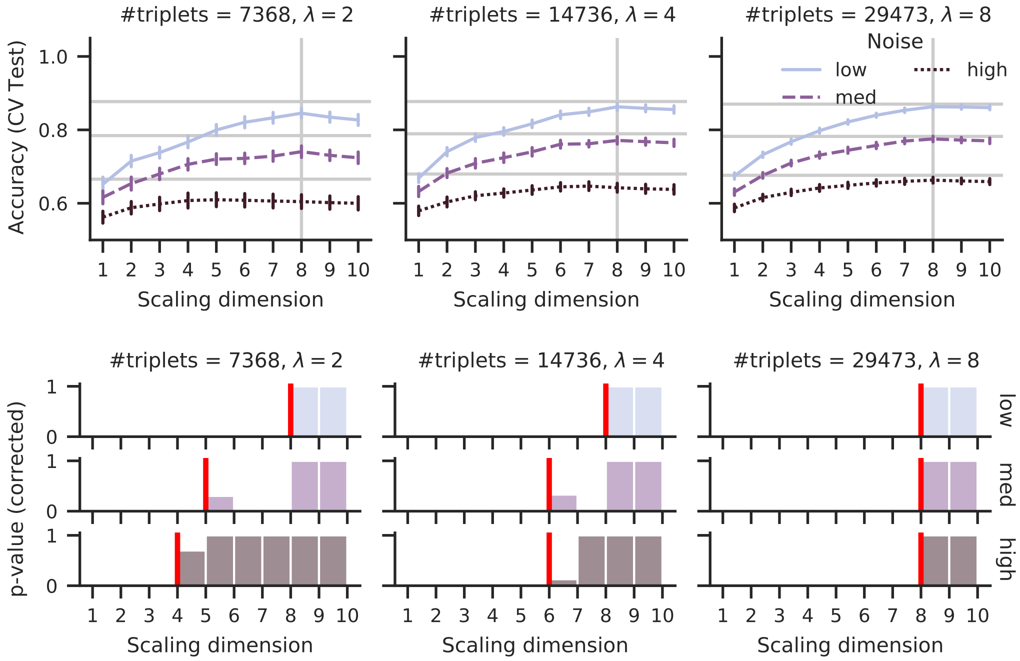

Figure 25. 8D-normal ($n = 100$)
